# Effect of Isoflavones on Blood Lipid Alterations in Postmenopausal Females: A Systematic Review and Meta-Analysis of Randomized Trials

**DOI:** 10.1016/j.advnut.2023.09.008

**Published:** 2023-09-25

**Authors:** Shengmin Yang, Qingjia Zeng, Xiaohong Huang, Zhen Liang, Hongpu Hu

**Affiliations:** 1 Department of Endocrinology, Key Laboratory of Endocrinology of National Health Commission, State Key Laboratory of Complex Severe and Rare Diseases, Peking Union Medical College Hospital, Chinese Academy of Medical Science and Peking Union Medical College, Beijing, China; 2 Institute of Medical Information/Medical Library, Chinese Academy of Medical Science & Peking Union Medical College, Beijing, China; 3 Chinese Academy of Medical Science and Peking Union Medical College, Beijing, China; 4 Department of Urology, Peking Union Medical College Hospital, Chinese Academy of Medical Science and Peking Union Medical College, Beijing, China

**Keywords:** isoflavones, postmenopausal females, lipids, meta-analysis, hormone replacement therapy

## Abstract

The effects of isoflavones on postmenopausal female’s blood lipid profile have yielded conflicting results in previous studies. Further investigation is necessary to determine the potential benefits of isoflavone therapy in managing cardiovascular health in this population.

This meta-analysis aimed to assess the effects of isoflavones on blood lipid concentrations in postmenopausal females. A comprehensive search was conducted in major databases for randomized controlled trials published between 2000 and 2023. Eighteen studies were included in the analysis, which examined the impact of isoflavone intake on blood lipids in postmenopausal females. Isoflavone consumption resulted in a significant reduction in triacylglycerol (TG) concentrations (–12.50 mg/dL; 95% CI: –23.09, –1.91) and a modest increase in high-density lipoprotein cholesterol (HDL cholesterol) concentrations (1.83 mg/dL; 95% CI: 0.03, 3.64). Subgroup analysis showed that isoflavones significantly decreased TG (–15.79 mg/dL; 95% CI: –28.36, –3.22) and increased HDL cholesterol (2.49 mg/dL; 95% CI: 1.80, 3.19) in postmenopausal females under 65 y old. No significant effects were observed in females over 65 y old. Both low (≤80 mg/d) and high (>80 mg/d) doses of isoflavones exhibited TG-lowering effects, whereas only the high dose increased HDL cholesterol. Longer treatment duration (≥24 wk) was associated with a significant reduction in TG, whereas HDL cholesterol improvement occurred during the early period (<24 wk) of supplementation. The consumption of isoflavones resulted in a significant reduction in TG concentrations and an increase in HDL cholesterol concentrations among postmenopausal females under 65 y of age.

**Statements of significance**

This meta-analysis is the most comprehensive evaluation to date, highlighting the differential impacts of isoflavone consumption on blood lipids in postmenopausal females based on age and dosage. Our findings emphasize that the consumption of isoflavones resulted in a significant reduction in TG concentrations and an increase in HDL cholesterol concentrations, especially in postmenopausal females below 65 y.

## Introduction

The incidence of cardiovascular disease is significantly lower in pre-menopausal females compared to males of the same age. However, after menopause, the incidence in females rapidly increases and approaches that of men. This temporal change suggests that female sex hormones may play a crucial role in protecting against atherosclerosis. Estrogen has been shown to reduce LDL cholesterol, increase HDL cholesterol, improve vascular function, and contribute to longer life expectancy in females [1,2]. Nevertheless, estrogen therapy in postmenopausal females has also been associated with an increased risk of specific side effects such as uterine and breast cancer [3,4].

The ideal hormone replacement therapy (HRT) should replicate the beneficial effects of estrogen without inducing the aforementioned adverse reactions. This concept has led to the development of selective estrogen receptor modulators (SERMs), which exhibit mixed functional activity as agonists or antagonists of the estrogen receptor depending on the target tissue [5]. However, synthetic SERMs have been reported to elevate risk of venous thrombosis and arterial thrombotic events like stroke in postmenopausal females [[6], [7], [8]]. In pursuing an effective HRT with improved safety and tolerability for postmenopausal females, natural SERMs have garnered significant interest. Epidemiological studies have revealed that the mortality rate from coronary heart disease is notably higher among American females than Japanese females. Furthermore, the incidence of coronary heart disease in Asian immigrants who maintained their traditional dietary habits was lower than in those who adopted a Westernized diet, with the difference primarily attributed to the high consumption of soy foods in Asian diets [9].

Isoflavones, the primary active compounds in soy foods, structurally resemble endogenous estrogen, bind to the estrogen receptor, and function as natural SERMs. Research has suggested that isoflavones may reduce risk of certain cancers, including lung, prostate, colon (in females only), and breast cancers, without any reported associations with thrombosis or stroke [10,11]. Thus, isoflavones hold promise as a potential therapeutic option for HRT in postmenopausal females. Previous studies have demonstrated positive effects of isoflavones on lipid parameters, such as reducing serum total cholesterol (TC), LDL cholesterol, and triacylglycerol (TG), as well as increasing HDL cholesterol [12,13]. However, clinical trials in postmenopausal females have yielded inconsistent results. Sex differences in plasma lipid responses to soy protein-containing isoflavones have been observed, with soy intake being negatively correlated with TC and LDL cholesterol in males and young females but not in females over 50 y old [14,15].

In this meta-analysis, we aimed to combine the findings from multiple studies, varying in sample sizes, to gain a deeper understanding of the effects of isoflavones on the changes in blood lipid concentrations in postmenopausal females.

## Methods

### Identification and selection of studies

Medline, Embase, and the Cochrane Central Register of Controlled Trials were thoroughly searched for English-language reports of randomized controlled trials (RCTs) published between 2000 and 2023, examining the effects of isoflavones on blood lipid profile. Search strategies were developed focusing on postmenopausal females, isoflavones, blood lipids, and RCTs (Supplemental Table 1). The inclusion criteria are as follows: *1*) the study population consisted exclusively of postmenopausal females; *2*) the study was an RCT with either a parallel or a crossover design; and *3*) the study provided information on the age of participants, as well as the dose and duration of isoflavone treatments. To ensure the credibility of the analysis, studies were excluded if they met any of the following criteria: *1*) the study was not in English; *2*) the research utilized unpurified isoflavones for treatment; *3*) the study lacked the required information or employed an inappropriate control group; and *4*) the study lacked follow-up data.

### Data extraction and quality assessment

Data were extracted independently by 2 authors (SY and QZ). Any discrepancies were resolved through discussion or by consulting a senior researcher (ZL). The extracted data included various study characteristics, such as the first author’s name, year of publication, study design, age and number of participants, dose and duration of isoflavones treatment, health characteristics of the study population, and location of the study. Additionally, information on the final concentrations of plasma/serum TC, LDL cholesterol, HDL cholesterol, and TG in the treatment and control group were also collected. The quality of the studies was assessed using risk of bias tool outlined in the Cochrane Handbook, which evaluated various aspects such as blinding and outcome reporting (Supplemental Figures 1 and 2)

### Data analysis

We conducted the meta-analysis using REVMAN version 5.2 (Cochrane Collaboration). The effect size employed in this study was the difference in means between the treatment and control groups. We utilized plasma/serum lipid concentrations obtained at the end of each intervention. If multiple time points for follow-up were reported, we included the value corresponding to the time point with the longest duration of follow-up. Typically, serum cholesterol concentrations are ∼3% higher than corresponding plasma concentrations [16]. However, for the purpose of analyzing mean differences in each study, we analyzed serum and plasma concentrations without adjusting for this difference.

For studies reporting results in mmol/L, we converted them to mg/dL using standard conversion factors (dividing the mmol/L value by 0.02586 for TC, LDL, and HDL and by 0.01129 for TG). Mean differences and their 95% confidence intervals (CIs) were calculated using random-effect or fixed-effect models. Heterogeneity was assessed using the χ^2^ test and the I^2^ statistics. If the test for heterogeneity yielded a significant result (I^2^ >30%), we presented the results from the random-effect models. Otherwise, the estimate of the difference was calculated using the fixed-effect model. To examine potential publication bias, we employed a funnel plot and conducted Egger’s regression asymmetry test. A 2-sided *P* value <0.05 was considered statistically significant.

## Results

### Identification and selection

**FIGURE 1:**
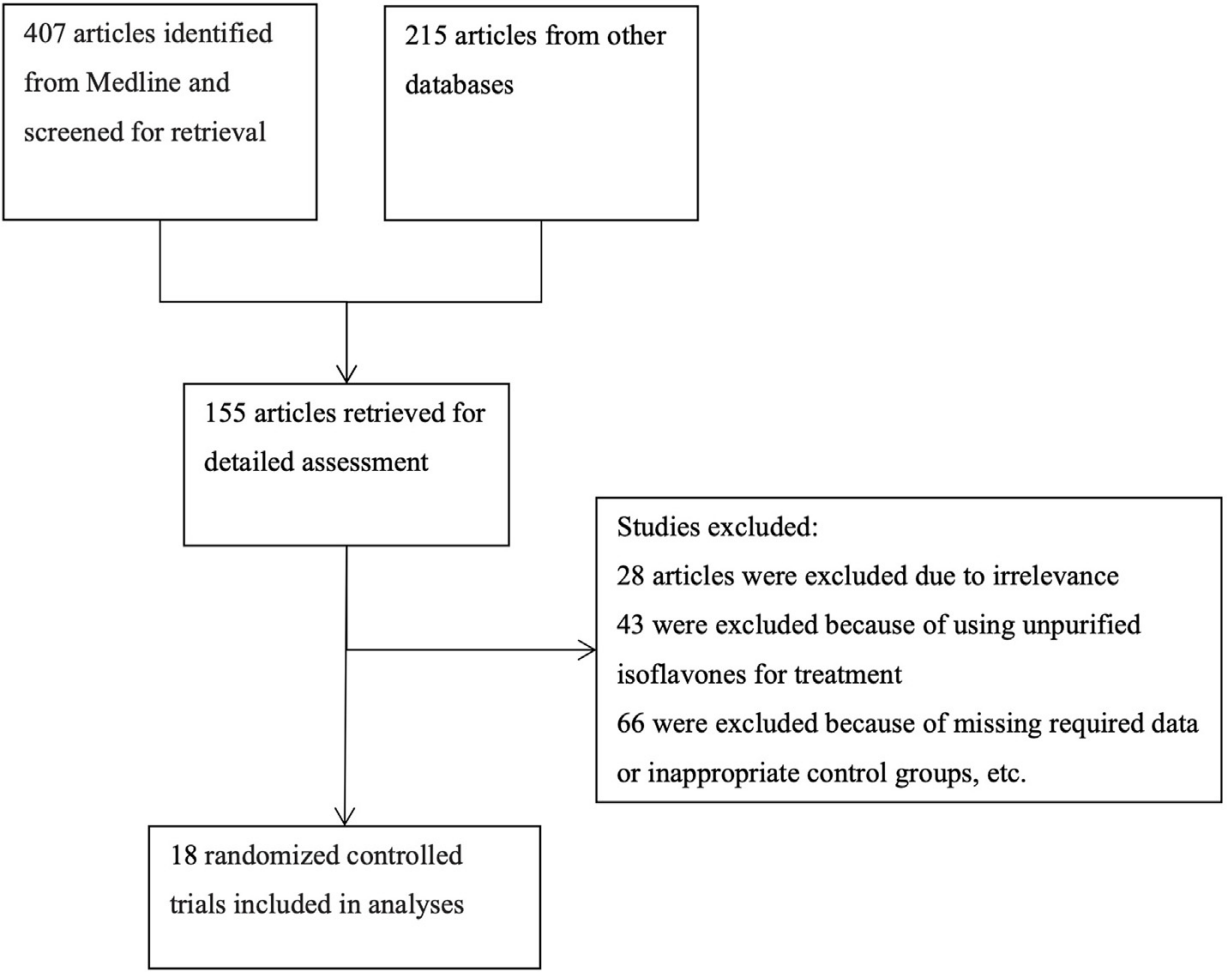
Study selection process.

Out of the initial 562 potentially relevant studies identified through database searches, a total of 18 articles comprising 20 comparisons met our inclusion criteria [[17], [18], [19], [20], [21], [22], [23], [24], [25], [26], [27], [28], [29], [30], [31], [32], [33], [34]]. A flowchart illustrating the selection process is presented in Figure 1.

### Study characteristics

The characteristics of the 18 studies that met the selection criteria are presented in Table 1. Among these studies, 9 focused exclusively on postmenopausal females under 65 y old [22,[24], [25], [26],^29^,[31], [32], [33], [34]], whereas the remaining 9 trials included older females (over 65 y old) [[17], [18], [19], [20], [21],^23^,^27^,^29^,^30^]. The trials varied in terms of isoflavone concentrations, ranging from 40 mg/d to 150 mg/d, and duration, ranging from 4 to 48 wk. Most of the RCTs were double-blinded, employed an appropriate method for sequence generation, and provided details regarding dropouts.

**TABLE 1:** Characteristics of the 18 studies, including 20 comparisons

Author	Year	Design	Age (y old)	Treat/control	Duration (wk)	Dose (mg/d)
G.R.R. Barrasa [17]	2018	Parallel	55–72	20/15	12	100
H. Braxas [18]	2019	Parallel	47–69	28/26	12	54
A. Dewell [19]	2002	Parallel	69 ± 4	20/16	24	150
A.K. Engelbert [20]	2016	Parallel	≤75	85/85	12	117.4
C. Gardner [21]	2001	Parallel	<80	31/33	12	80
A. Garrido [22]	2006	Parallel	45–60	15/14	12	100
J.S. Giolo [23]	2018	Parallel	50–70	17/15	10	100
S.C. Ho [24]	2007	Parallel	48–62	68/68 67/68	48	40 80
H.K. Jassi [25]	2010	Parallel	40–60	25/25	12	60
J. Kim [26]	2013	Parallel	50–57	42/43	12	70
M. Leheudre [27]	2007	Parallel	50–70	10/10	48	70
Z.M. Liu [28]	2012	Parallel	46–70	60/60	24	100
Z.M. Liu [29]	2014	Parallel	48–65	90/90	24	63
D.R. Rios [30]	2008	Parallel	47–66	25/22	24	40
T. Uesugi [31]	2002	Parallel	40–62	12/11	4	61.8
J. Wu [32]	2006	Parallel	45–60	33/33	48	75
Y. Ye [33]	2012	Parallel	45–60	26/27 25/27	24 24	84 126
T. Zhang [34]	2019	Parallel	52–62	77/83	24	60

### Changes in blood lipid concentrations

**FIGURE 2:**
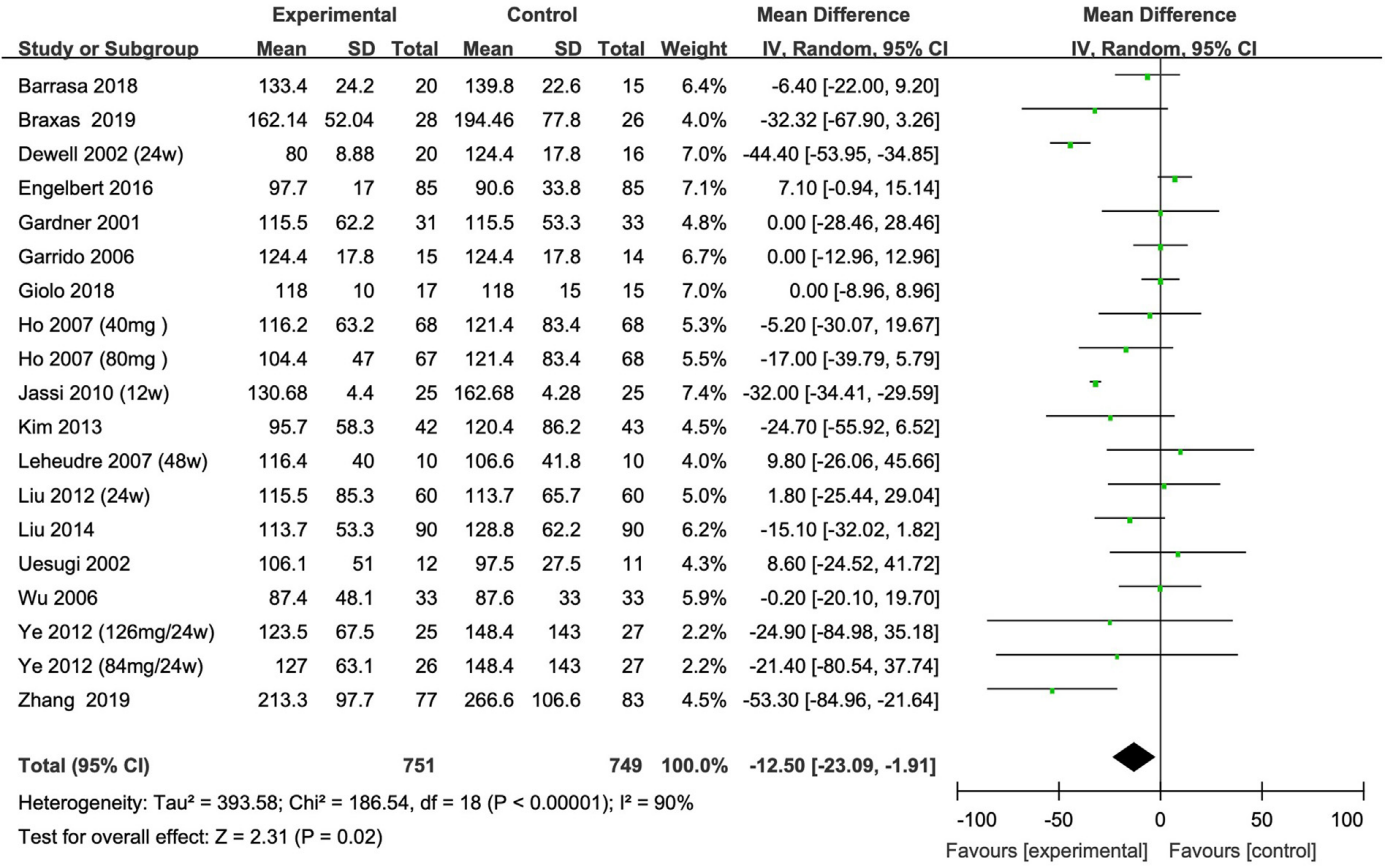
Meta-analysis of the effect of isoflavone on TG in all the postmenopausal women involved in the study. The sizes of the data markers indicate the weight of each study in the analysis. Values are in mg/dL. CI, confidence interval; IV, inverse variance; Random, random-effect model; SD, standard deviation; TG, triacylglycerol.

**FIGURE 3:**
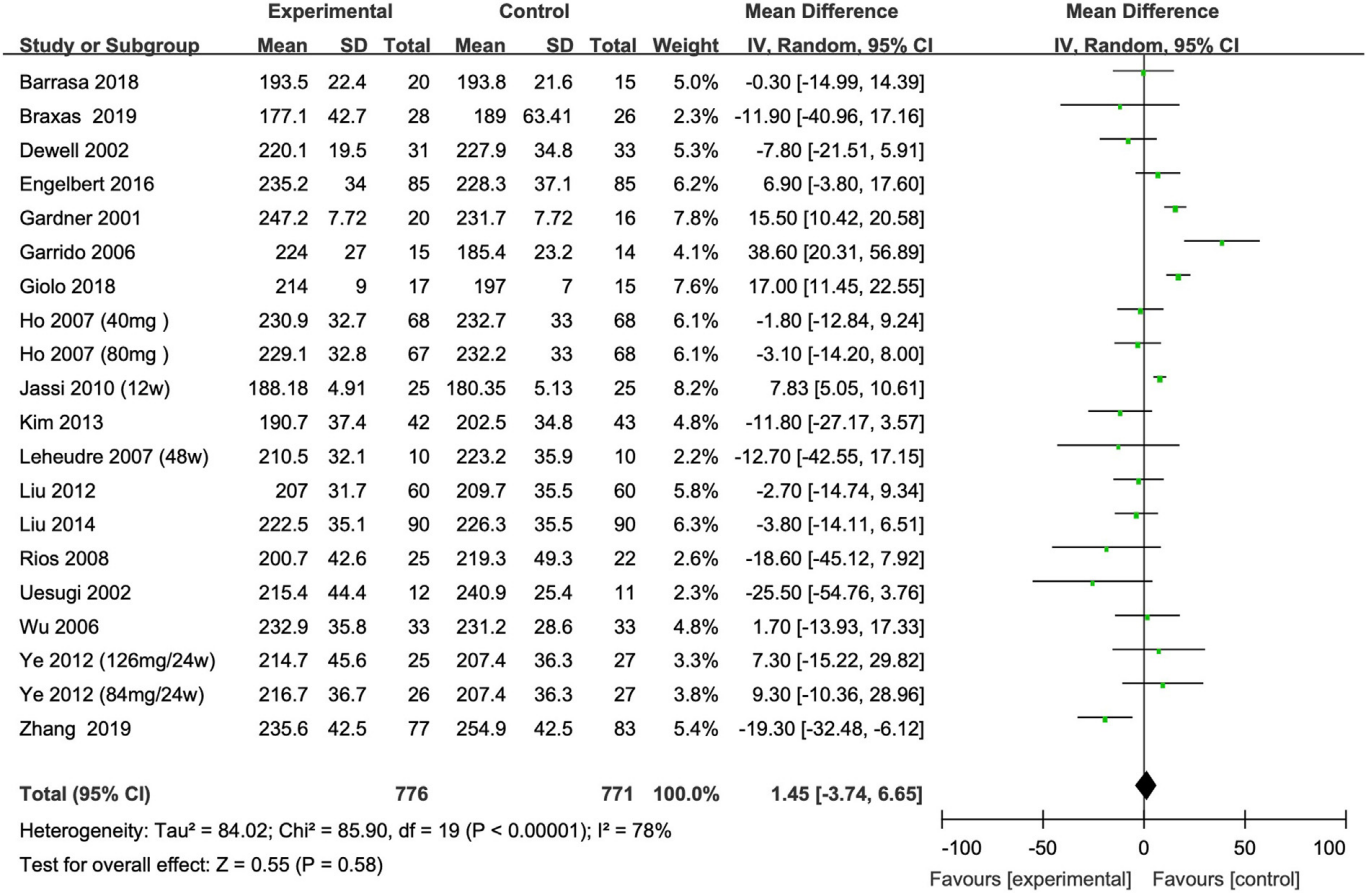
Meta-analysis of the effect of isoflavone on TC in all the postmenopausal women involved in the study. The sizes of the data markers indicate the weight of each study in the analysis. Values are in mg/dL. CI, confidence interval; IV, inverse variance; Random, random-effect model; SD, standard deviation; TC, total cholesterol.

**FIGURE 4:**
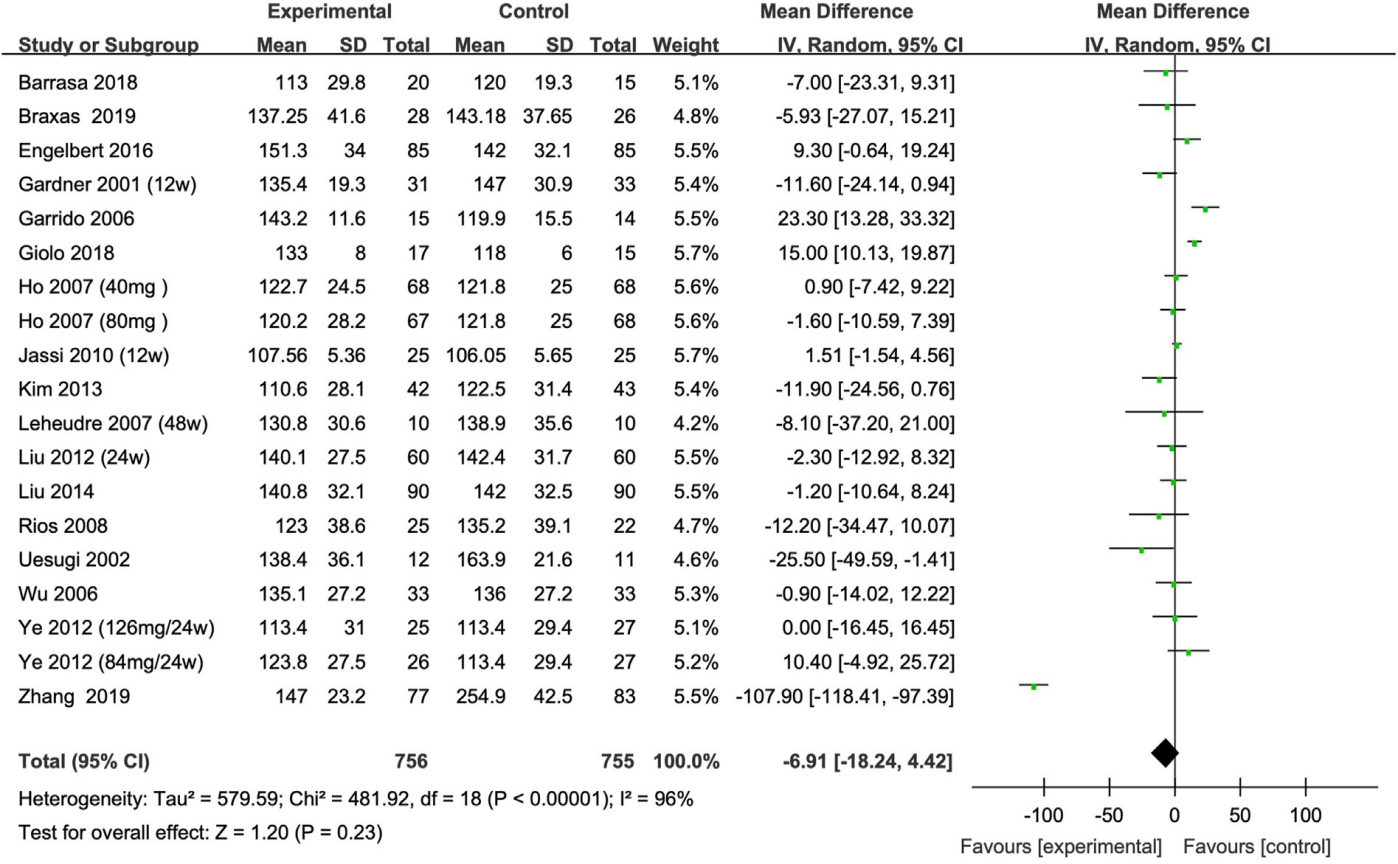
Meta-analysis of the effect of isoflavone on LDL cholesterol in all the postmenopausal women involved in the study. The sizes of the data markers indicate the weight of each study in the analysis. Values are in mg/dL. CI, confidence interval; IV, inverse variance; LDL cholesterol, low-density lipoprotein cholesterol; Random, random-effect model; SD, standard deviation.

**FIGURE 5:**
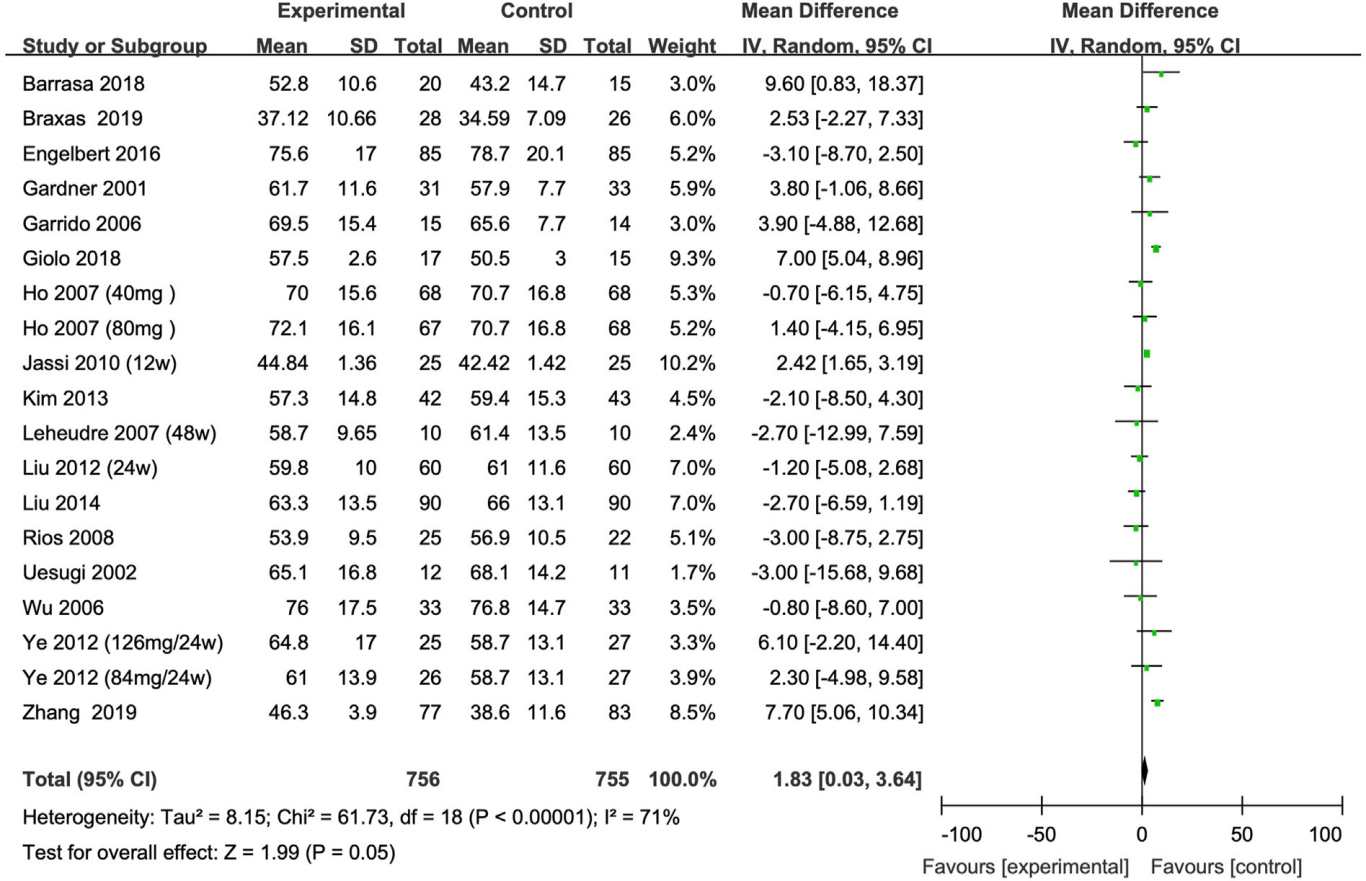
Meta-analysis of the effect of isoflavone on HDL cholesterol in all the postmenopausal women involved in the study. The sizes of the data markers indicate the weight of each study in the analysis. Values are in mg/dL. CI, confidence interval; HDL cholesterol, high-density lipoprotein cholesterol; IV, inverse variance; Random, random-effect model; SD, standard deviation.

Intake of isoflavones resulted in a significant reduction in blood TG concentration, with a mean difference of –12.50 mg/dL (95% CI: –23.09, –1.91), as shown in Figure 2. Additionally, a modest increase in HDL cholesterol was observed, with a mean difference of 1.83 mg/dL (95% CI: 0.03, 3.64), as shown in Figure 3. However, no significant changes were found in TC, with a mean difference of 1.45 mg/dL (95% CI: –3.74, 6.65) as shown in Figure 4, and in LDL cholesterol, with a mean difference of –6.91 mg/dL (95% CI: –18.24, 4.42) as shown in Figure 5.

### Subgroup analyses

In the sub-studies involving only postmenopausal females under 65 y old, there was a significant reduction in TG concentrations, with a mean difference of –15.79 mg/dL (95% CI: –28.36, –3.22) and an increase in HDL cholesterol concentrations, with a mean difference of 2.49 mg/dL (95% CI: 1.80, 3.19). However, changes in TC concentrations, with a mean difference of 0.33 mg/dL (95% CI: –7.39, 8.05), and LDL cholesterol concentrations, with a mean difference of –4.96 mg/dL (95% CI: –14.62, 4.70), remained non-significant. In the sub-studies that included postmenopausal females over 65 y old, none of the results for TG, TC, LDL cholesterol, and HDL cholesterol were significant (Table 2). The pooled estimates of treatment effects on lipid profiles in the defined subgroups of trials, based on the dose and intervention time of isoflavones, are summarized in TABLE 3, TABLE 4, respectively.

**TABLE 2:** Effect of isoflavones on blood lipids by age (≤65 y old or >65 y old)

Lipids	Number of comparisons	Sample size (experimental/control)	Mean difference (mg/dL)	95% CI (mg/dL)	*P* value
Age was limited to be ≤65 y old					
TG	11	480/489	–15.79	–28.36, –3.22	0.01
TC	11	480/489	0.33	–7.39, 8.05	0.08
LDL cholesterol	11	480/489	–4.96	–14.62, 4.70	0.31
HDL cholesterol	11	480/489	2.49	1.80, 3.19	<0.0001
Age was limited to be >65 y old					
TG	8	271/260	–10.2	–27.43, 7.03	0.25
TC	9	296/282	2.84	–4.98, 10.66	0.48
LDL cholesterol	8	276/266	–0.85	–10.21, 8.42	0.85
HDL cholesterol	8	276/266	–2.15	–1.35, 5.66	0.23

CI, confidence interval; HDL cholesterol, high-density lipoprotein cholesterol; LDL cholesterol, low-density lipoprotein cholesterol; TC, total cholesterol; TG, triacylglycerol.

**TABLE 3:** Effect of isoflavones on blood lipids by dose (≤80 mg/d or >80 mg/d)

Lipids	Number of comparisons	Sample size (experimental/control)	Mean difference (mg/dL)	95% CI (mg/dL)	*P* value
Dose was limited to be ≤80 mg/d					
TG	11	483/490	–16.9	–28.11, –5.69	0.003
TC	12	508/512	–6.02	–13.26, 0.86	0.09
LDL cholesterol	12	508/512	–4.50	–9.36, –1.64	0.12
HDL cholesterol	12	508/512	1.21	–0.90, 3.27	0.23
Dose was limited to be >80 mg/d					
TG	8	268/259	–9.11	–26.0, 3.77	0.04
TC	8	268/259	6.42	4.67, 8.17	0.09
LDL cholesterol	7	248/243	4.25	0.73, 5.78	0.13
HDL cholesterol	7	248/243	3.19	0.80, 7.19	0.02

CI, confidence interval; HDL cholesterol, high-density lipoprotein cholesterol; LDL cholesterol, low-density lipoprotein cholesterol; TC, total cholesterol; TG, triacylglycerol.

**TABLE 4:** Effect of isoflavones on blood lipids by duration (<24 wk or ≥24 wk)

Lipids	Number of comparisons	Sample size (experimental/control)	Mean difference (mg/dL)	95% CI (mg/dL)	*P* value
Duration was limited to be <24 wk					
TG	8	250/242	–8.33	–24.55, 7.89	0.31
TC	9	275/267	4.82	–2.80, 12.45	0.22
LDL cholesterol	9	275/267	1.09	–6.93, 9.12	0.79
HDL cholesterol	9	275/267	2.85	0.38, 5.32	0.02
Duration was limited to be ≥24 wk					
TG	10	476/482	–18.73	–33.52, –3.94	0.01
TC	11	501/504	4.58	–1.22, 7.95	0.08
LDL cholesterol	10	481/488	–7.17	–18.62, 4.29	0.22
HDL cholesterol	10	481/488	0.78	–2.40, 3.95	0.63

CI, confidence interval; HDL cholesterol, high-density lipoprotein cholesterol; LDL cholesterol, low-density lipoprotein cholesterol; TC, total cholesterol; TG, triacylglycerol.

When the limit of isoflavones ingestion was set at ≤80 mg/d and >80 mg/d, significant reductions in TG were observed in the isoflavones groups compared to the corresponding control groups for both the ≤80 mg/d and >80 mg/d subjects. Additionally, when ingesting isoflavones exceeded 80 mg/d, there was a significant increase in HDL cholesterol (Table 3).

In terms of the duration of isoflavone intake, no significant changes in TG were found in the isoflavone group compared to the corresponding control group for subjects with a duration of <24 wk. However, when the duration exceeded 24 wk, there was a significant decrease in TG. On the contrary, HDL cholesterol concentrations increased significantly in studies with a duration of <24 wk of isoflavone intake (Table 4).

### Changes of heterogeneities

As for heterogeneity, the I^2^ values ranged from 71% to 96% in the overall analysis. In the subgroup analyses based on age, dose, and intervention time of isoflavones, the I^2^ values ranged from 64% to 91%, 65% to 91%, and 72% to 95%, respectively.

### Publication bias

**FIGURE 6:**
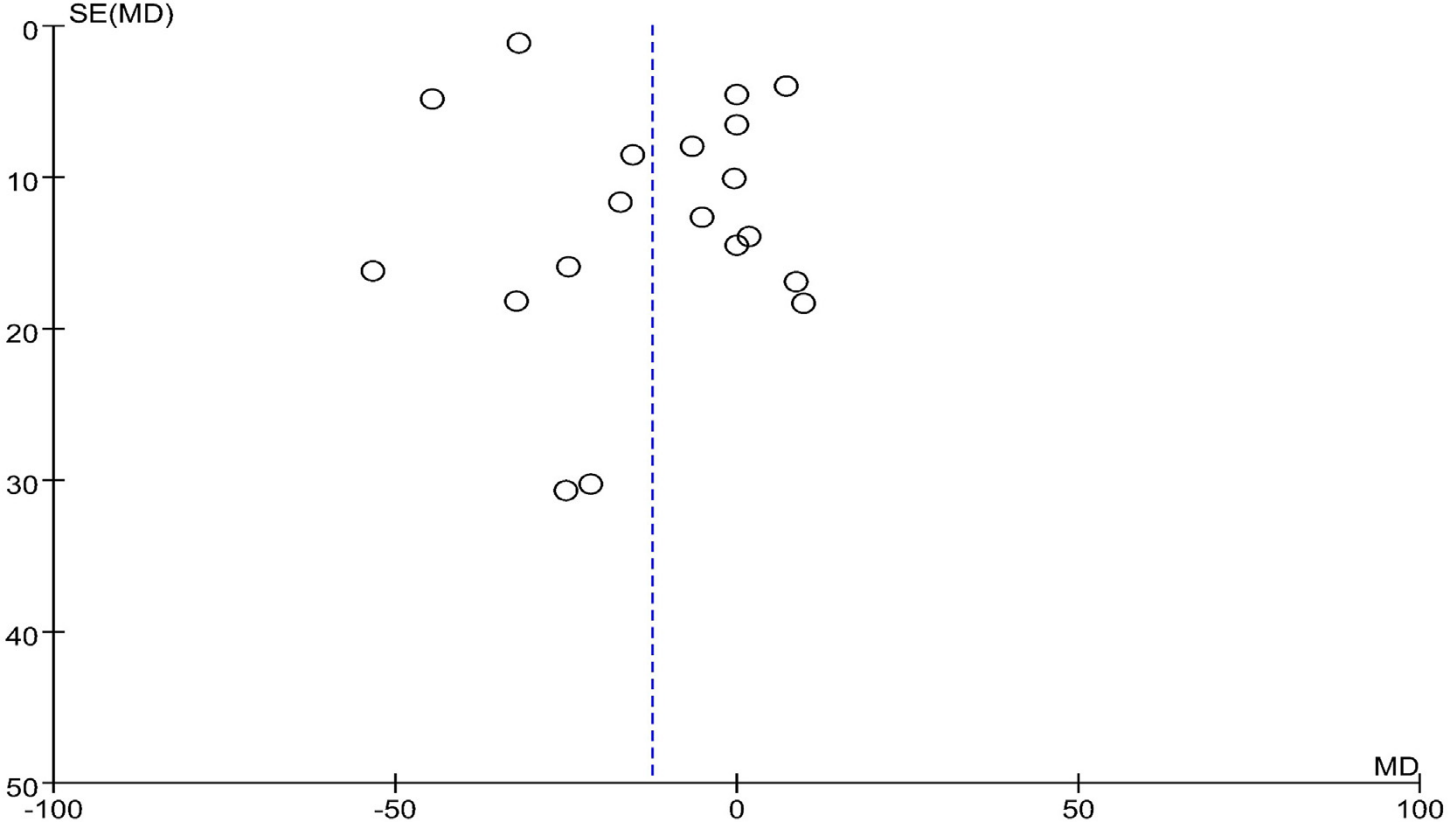
Examination of publication bias about TG on the basis of a funnel plot in all the postmenopausal women involved in the study, which plotted the SEM of the studies against their corresponding effect sizes. MD, mean difference; SEM, standard error of the mean; TG, triacylglycerol.

**FIGURE 7:**
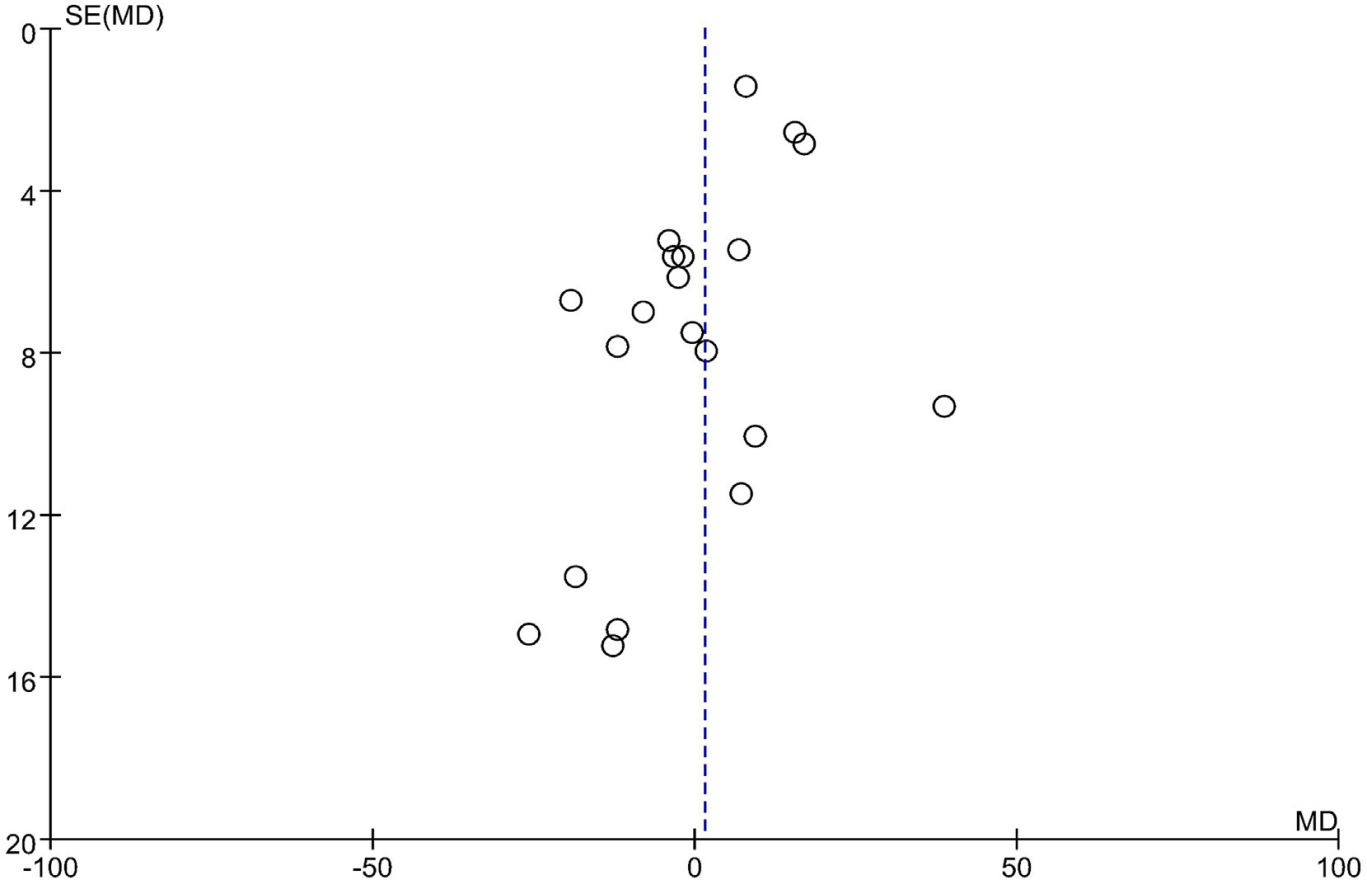
Examination of publication bias about TC on the basis of a funnel plot in all the postmenopausal women involved in the study, which plotted the SEM of the studies against their corresponding effect sizes. MD, mean difference; SEM, standard error of the mean; TC, total cholesterol.

**FIGURE 8:**
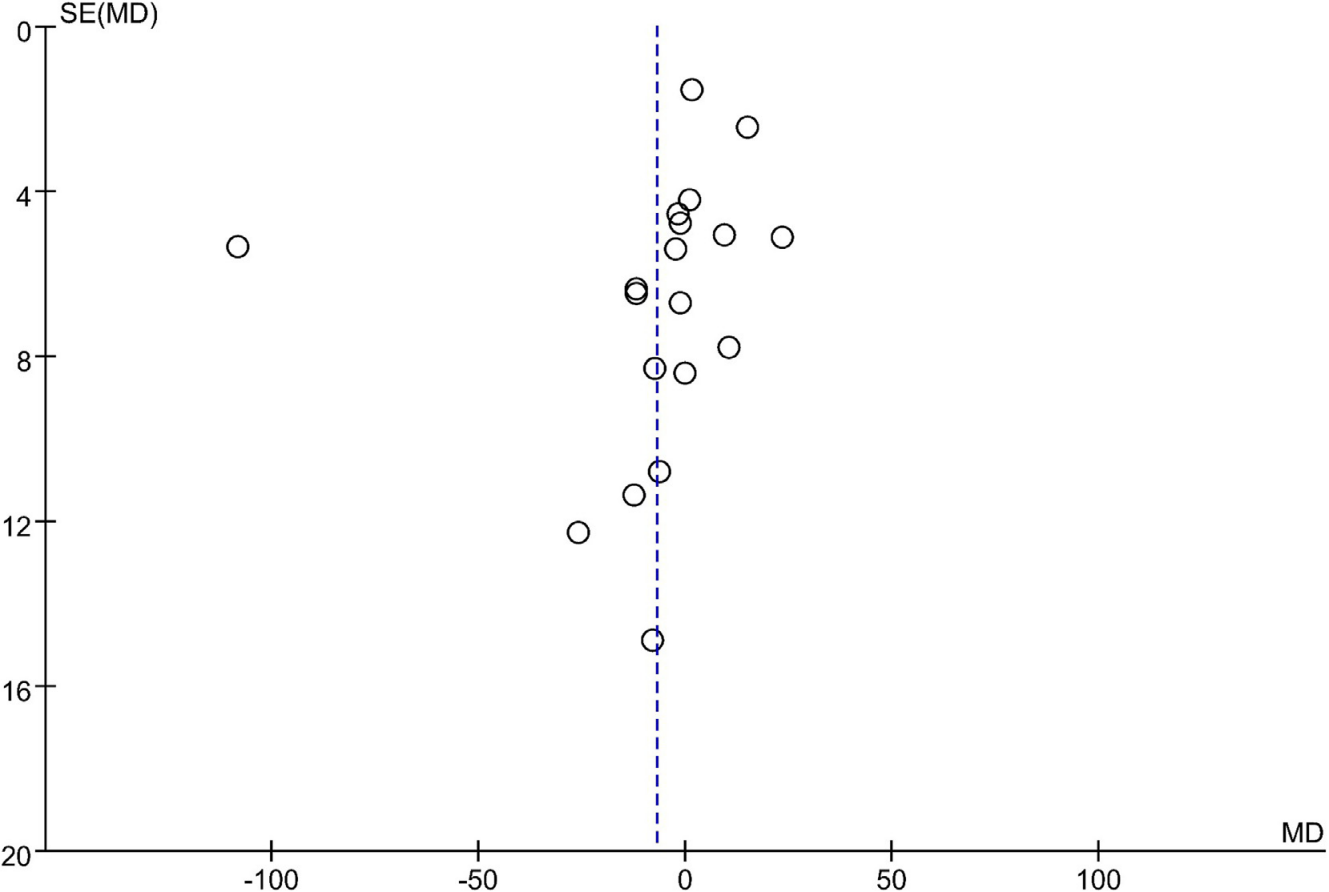
Examination of publication bias about LDL cholesterol on the basis of a funnel plot in all the postmenopausal women involved in the study, which plotted the SEM of the studies against their corresponding effect sizes. LDL cholesterol, low-density lipoprotein cholesterol; MD, mean difference; SEM, standard error of the mean.

**FIGURE 9:**
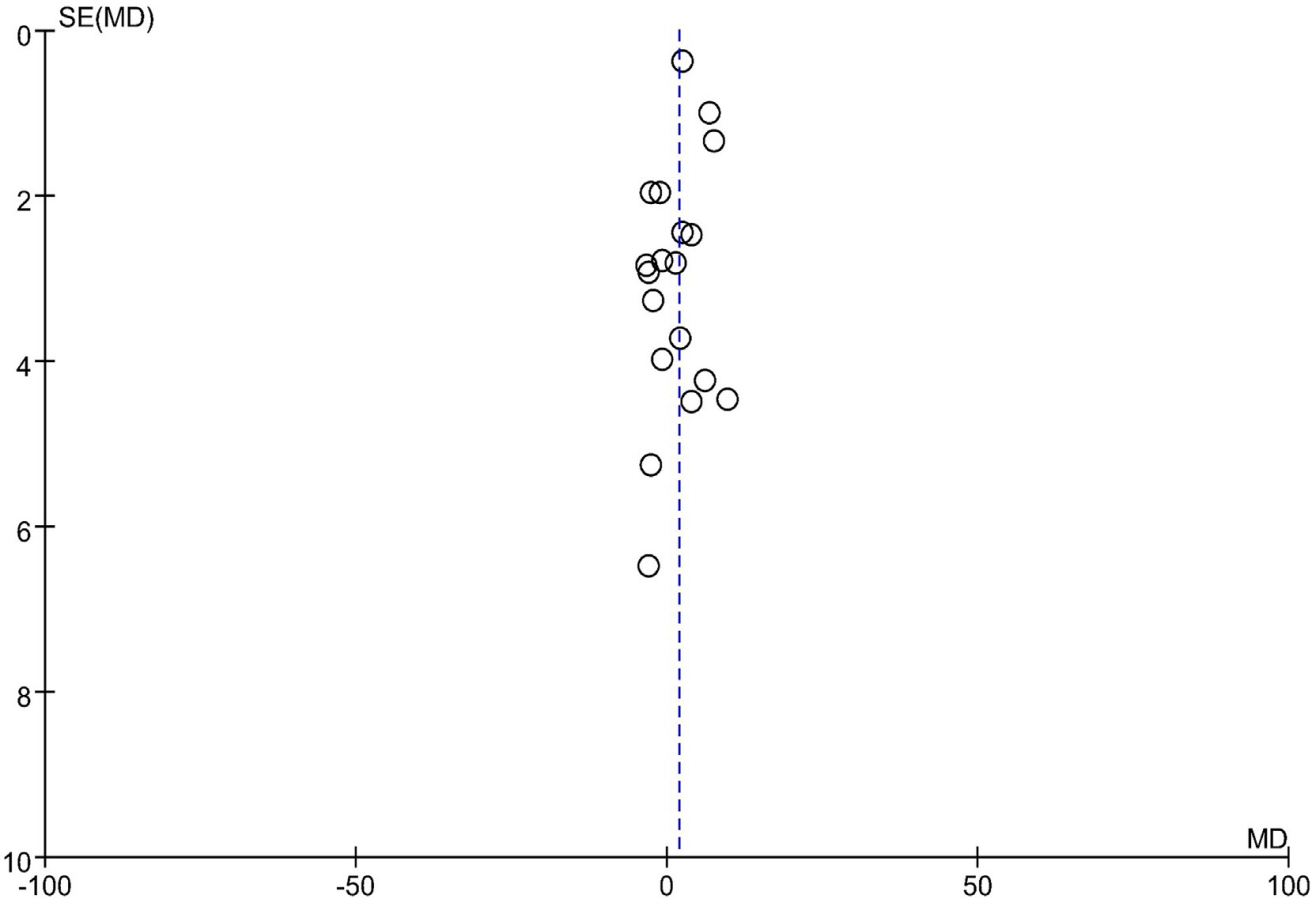
Examination of publication bias about HDL cholesterol on the basis of a funnel plot in all the postmenopausal women involved in the study, which plotted the SEM of the studies against their corresponding effect sizes. HDL cholesterol, high-density lipoprotein cholesterol; MD, mean difference; SEM, standard error of the mean.

The funnel plots depicting the effects on lipid profile in the comparison groups are presented in FIGURE 6, FIGURE 7, FIGURE 8, FIGURE 9. To assess the presence of publication bias for blood TG, TC, LDL cholesterol, and HDL cholesterol concentrations, Egger’s test and the trim and fill method were utilized. However, no significant differences indicative of publication bias were observed.

## Discussion

Although numerous clinical trials have investigated the effects of isoflavones on blood lipid concentrations in postmenopausal females, the results have been inconsistent. The present study quantitatively analyzed RCTs conducted in the past 23 y that specifically focused on the effects of isoflavones on lipid profiles in postmenopausal females.

Our analysis demonstrates that the intake of isoflavones is associated with favorable effects on blood lipids in postmenopausal females. Specifically, we observed a significant reduction in TG and an increase in HDL cholesterol among postmenopausal females under 65 y old. However, no significant effects were observed in older participants. These findings suggest that isoflavone supplementation may significantly benefit the lipid profile by decreasing TG and increasing HDL cholesterol, specifically in postmenopausal females under 65 y old. Subgroup analysis further revealed that remarkable reductions in TG were observed in the isoflavones groups across different doses (≤80 mg/d and >80 mg/d) and intervention durations (≥24 wk). Moreover, HDL cholesterol significantly increased in studies where isoflavone intake exceeded 80 mg/d or had a duration of <24 wk.

Numerous studies have provided evidence supporting the significant role of blood lipids in developing cardiovascular disease. Research has shown that LDL cholesterol is the primary risk factor for atherosclerosis [35]. Small dense LDL cholesterol particles, in particular, pose greater harm to cardiovascular health because of their stronger affinity for artery intimal proteoglycans, making it easier for them to penetrate the vascular intima. Additionally, these small, dense particles are more prone to oxidation and have slower clearance from circulation compared to larger particles [36]. Moreover, small dense LDL cholesterol particles contain higher concentrations of cholesterol and TG, and increased TG concentration has been positively linked to the presence of small dense LDL cholesterol, confirming hypertriglyceridemia as an independent risk factor for cardiovascular disease [37]. On the contrary, HDL cholesterol, often referred to as “good cholesterol,” offers cardiovascular benefits, and higher HDL cholesterol has been associated with a decreased risk of myocardial infarction-related morbidity [38].

The mechanisms underlying the effects of isoflavones on lipid metabolism are still being investigated. One possible explanation is their weak estrogenic effects. Previous research has indicated that isoflavones can enhance the activity of steroidogenic enzymes such as cytochrome P450 aromatase, 3β-Hydroxysteroid dehydrogenase, and 17β-hydroxysteroid dehydrogenase, leading to increased estrogen secretion [39,40]. Isoflavones have also been shown to stimulate the synthesis of sex hormone-binding globulin, reducing estrogen clearance [41,42]. In addition to these weak estrogenic effects, isoflavones may influence serum lipids through other mechanisms, such as reducing lipid absorption and increasing the excretion of steroids in feces [43]. Therefore, it is important to note that the effect of isoflavones on blood lipids is not identical to that of estrogen. Estrogen has dual effects on lipids, including beneficial effects such as decreasing serum TC and LDL cholesterol while increasing HDL cholesterol. However, estrogen is also known to elevate serum TG concentrations by inhibiting the clearance of chylomicron emulsion and VLDL cholesterol. Based on the findings of this meta-analysis, it appears that isoflavones may be more effective than estrogen in counteracting the unfavorable changes in TG concentrations in postmenopausal females. However, the favorable effects of isoflavones on blood cholesterol concentrations may be weaker compared to estrogen.

Studies have shown that the impact of estrogen on lipid variations depends on age [44,45]. A recent study suggested that isoflavone supplementation was associated with favorable effects on the lipid profile in postmenopausal females under 65 y of age, but not in females over 65 y old [17]. Nevertheless, it should be noted that the number of participants in the experimental groups was relatively low (*n* = 11 for females under 65 y old, *n* = 9 for females over 65 y old). To further explore whether the effects of isoflavones are truly associated with the age of postmenopausal females, a subgroup meta-analysis was conducted. The results indicated that the effects of isoflavones on lipid metabolism were only significant when the participants were under 65 y old. This suggests that initiating isoflavone supplementation early may be more effective in counteracting the unfavorable changes in blood lipids that occur after menopause. The specific processes underlying the age-related impact of isoflavones on blood lipids remain unknown. Estrogen therapy has been found to have favorable effects on cardiovascular disease risk factors when initiated near the time of menopause [44,45], suggesting that isoflavones, acting as natural SERMs, may influence lipid metabolism through a mechanism related to estrogen-like effects in an age-dependent manner.

A study conducted by Ho et al. [25] found that a daily dose of 80 mg of isoflavones may not be sufficient to counteract the significant increase in cholesterol that occurs after menopause. The findings of this meta-analysis indicate that the intake of isoflavones at a low dosage (≤80 mg/d) or high dosage (>80 mg/d) is strongly associated with a significant decrease in blood TG concentrations. However, an increase in HDL cholesterol concentrations was only observed in subjects taking a high dosage of isoflavones (>80 mg/d). These results suggest that a limited dose of isoflavones can reduce blood TG concentrations, and increasing the intake of isoflavones beyond the maximal effective dose is not beneficial. On the contrary, a high dose of isoflavones is necessary to improve HDL cholesterol concentrations. Furthermore, the reduction in TG concentrations was observed in the isoflavones group with a treatment duration of 24 wk or longer, whereas the increase in HDL cholesterol concentrations was significant in subjects with a treatment duration of <24 wk. These findings suggest that long-term intake of isoflavones is needed to reduce blood TG concentrations, whereas the increase in blood HDL cholesterol occurs during the early period of isoflavone treatment.

One limitation of this analysis is that it was based on data from studies investigating the effects of isoflavones, whereas it is likely to be more relevant to real-life situations where soy foods are included as part of the diet. Another limitation to consider is the exclusive inclusion of only English-language studies, which may have omitted relevant research from non-English sources. Additionally, considerable heterogeneity was noted in the treatment effects of the included studies. A major contributor to this heterogeneity appears to be the diverse geographical origins of the subjects, including China, Japan, Korea, Canada, America, Brazil, Chile, and Germany. Moreover, the action mechanisms by which isoflavones modify the lipid profile have not been fully established in the discussions. It is also noteworthy that the effect of isoflavones seems to be predominantly observed in the population under 65 y of age, though the reasons for this remain unclear. Furthermore, although most of the included RCTs recruited healthy postmenopausal females, some studies also included females with conditions such as hyperlipidemia [19,34], diabetes [18], and obesity [27].

In conclusion, this meta-analysis demonstrates a strong association between the consumption of isoflavones and notable reductions in blood TG concentrations, as well as increased concentrations of HDL cholesterol in postmenopausal females under 65 y of age, and when aiming to regulate lipid concentrations, it is advisable to consider the appropriate dosage and duration of isoflavone intake. Given the substantial demand for safe and effective HRT to enhance quality of life and life expectancy, numerous studies have focused on natural SERMs. Based on the findings of this meta-analysis, isoflavone therapy appears to be a valuable alternative to estrogen for postmenopausal females below 65 y old. However, additional research is necessary to validate the reported results, and a thorough exploration of the underlying mechanism is warranted.

### Author contributions

The authors’ responsibilities were as follows– SY: designed the study; SY and QZ: contributed to the interpretation and analysis of the data; SY, QZ, and ZL: wrote the initial draft; HH and XH contributed to the revision of the manuscript, and all authors: read and approved the final manuscript.

### Conflict of Interest

The authors declare the following financial interests/personal relationships which may be considered as potential competing interests: Hongpu Hu reports financial support was provided by National Social Science Fund of China.

### Funding

This research was funded by the National Social Science Fund of China (22&ZD141 and 22AZD089). The funding bodies were not involved in any aspect of the study, from its conception and design to the collection, handling, analysis, or interpretation of the data, nor in the drafting, reviewing, or approving the manuscript.

### Data availability

Data described in the manuscript, code book, and analytic code will be made publicly and freely available without restriction. Original data generated and analyzed during this study are included in this published article or the data repositories listed in References.

## Appendix A Supplementary data

Multimedia component1 Multimedia component1
