# Supplementary material for: Effect of Isoflavones on Blood Lipid Alterations in Postmenopausal Females: A Systematic Review and Meta-Analysis of Randomized Trials
Source: Adv Nutr. 2023 Sep 25;14(6):1633–43. doi: 10.1016/j.advnut.2023.09.008 (PMC10721517; doi:10.1016/j.advnut.2023.09.008)
Supplement: Multimedia component1 [file mmc1.docx]

**Effect of Isoflavones on Blood Lipid Alterations in Postmenopausal Women: A Systematic Review and Meta-Analysis of Randomized Trials**

Shengmin Yang, Qingjia Zeng

**Supplementary materials**

**Supplementary Table 1**. Detailed search strategy for Medline

| **#** | **Term** |
| --- | --- |
| #1 | postmenopausal |
| #2 | post-menopause |
| #3 | elderly |
| #4 | aged |
| #5 | #1 OR #2 OR #3 OR #4 |
| #6 | isoflavones |
| #7 | phytoestrogen |
| #8 | soy |
| #9 | #6 OR #7 OR #8 |
| #10 | women |
| #11 | female |
| #12 | #10 OR #11 |
| #13 | #5 AND #12 |
| #14 | treat* |
| #15 | therapy |
| #16 | supplement |
| #17 | intake |
| #18 | #14 OR #15 OR #16 OR #17 |
| #19 | #9 AND #18 |
| #20 | “Lipids”[Mesh] |
| #21 | “Fats”[Mesh] |
| #22 | “Hyperlipidemia”[Mesh] |
| #23 | “Dyslipidemia”[Mesh] |
| #24 | metabolism |
| #25 | cholesterol |
| #26 | lipoprotein |
| #27 | LDL-C |
| #28 | #20 OR #21 OR #22 OR #23 OR #24 OR #25 OR #26 OR #27 |
| #29 | RCT |
| #30 | randomized controlled trial |
| #31 | #29 OR #30 |
| #32 | #13 AND #19 AND #28 AND #31 |


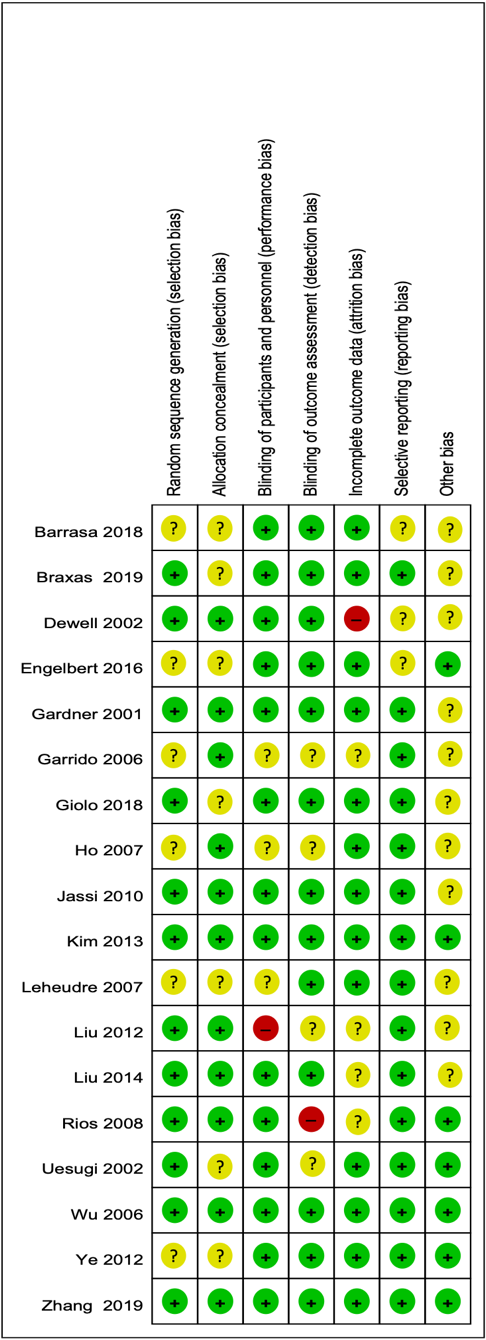


**Supplementary Figure 1.** Summary of Cochrane risk of bias for each study [16-33]


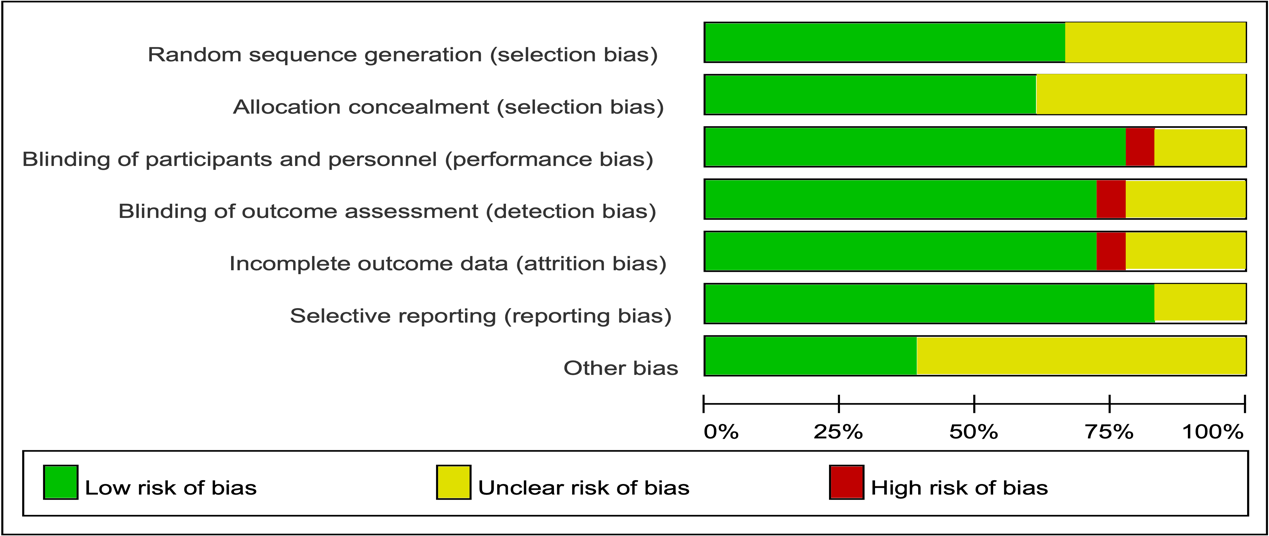


**Supplementary Figure 2.** Summary of risk of bias for each item presented as percentages for all studies included
